# Sol-Gel Derived Gelatin–Bioactive Glass Nanocomposite Biomaterials Incorporating Calcium Chloride and Calcium Ethoxide

**DOI:** 10.3390/polym16060747

**Published:** 2024-03-08

**Authors:** Rebeca Arambula-Maldonado, Kibret Mequanint

**Affiliations:** 1 School of Biomedical Engineering, University of Western Ontario, 1151 Richmond Street, London, ON N6A 5B9, Canada; rarambul@uwo.ca; 2 Department of Chemical and Biochemical Engineering, University of Western Ontario, 1151 Richmond Street, London, ON N6A 5B9, Canada

**Keywords:** sol-gel, calcium, gelatin–bioactive glass–MWCNT biomaterials, nanocomposites, bioactivity, bone biomaterial

## Abstract

Calcium-containing organic–inorganic nanocomposites play an essential role in developing bioactive bone biomaterials. Ideally, bone substitute materials should mimic the organic–inorganic composition of bone. In this study, the roles of calcium chloride (CaCl_2_) and calcium ethoxide (Ca(OEt)_2_) were evaluated for the development of sol-gel-derived organic–inorganic biomaterials composed of gelatin, bioactive glass (BG) and multiwall carbon nanotubes (MWCNTs) to create nanocomposites that mimic the elemental composition of bone. Nanocomposites composed of either CaCl_2_ or Ca(OEt)_2_ were chemically different but presented uniform elemental distribution. The role of calcium sources in the matrix of the nanocomposites played a major role in the swelling and degradation properties of biomaterials as a function of time, as well as the resulting porous properties of the nanocomposites. Regardless of the calcium source type, biomineralization in simulated body fluid and favorable cell attachment were promoted on the nanocomposites. 10T1/2 cell viability studies using standard media (DMEM with 5% FBS) and conditioned media showed that Ca(OEt)_2_-based nanocomposites seemed more favorable biomaterials. Collectively, our study demonstrated that CaCl_2_ and Ca(OEt)_2_ could be used to prepare sol-gel-derived gelatin–BG–MWCNT nanocomposites, which have the potential to function as bone biomaterials.

## 1. Introduction

Biomaterials that mimic the structural composition of bone tissue are desired for the repair or regeneration of fractures and defects. Native bone is composed of a complex hierarchical structure comprising collagen as the main organic component and hydroxycarbonate apatite as the inorganic component [1,2,3]. The selection of appropriate materials for the preparation of organic–inorganic nanocomposites that simulate the composition of bone is crucial.

Bioactive glasses (BG) are ideal for use as an inorganic component for the preparation of bone biomaterials since they readily bond to bone and stimulate both osteoconduction and osteoinduction [4,5]. BGs are amorphous, silicate-based materials that are based on a covalent random network of corner-sharing silica tetrahedra containing Si-O-Si bridging bonds [6]. The original BG (46.1% SiO_2_, 24.4% Na_2_O, 26.9% CaO, and 2.6% P_2_O_5_, in mol%), 45S5 Bioglass^®^, was developed by Hench and his colleagues [7]. Bioglass^®^ was first produced through melt-quenching processing which involved melting the oxide components in platinum crucibles at a temperature of 1370 °C, followed by pouring into a preheated mold or pouring the melt into water to quench, creating a powder or frit [7,8]. One of the potential ways bone bonds to BG is through the formation of a hydroxycarbonate apatite (HCA) layer on the surface of the glasses in contact with body fluid, similar to the apatite in bone, that would facilitate the formation of a strong bond through the plausible interaction of collagen fibrils from the host bone and the HCA nodules forming on the glass [7]. This process occurs through the release of soluble ionic species from the glass to form a high-surface-area hydrated silica and polycrystalline HCA bilayer on the glass surface [8].

BG can also be synthesized via the sol-gel process. Sol-gel-derived BGs are produced through the hydrolysis of alkoxide precursors to form a colloidal silica sol [9]. Metal alkoxides, such as tetraethyl orthosilicate (TEOS,) are commonly used as silica precursors due to their ability to readily react with water [10]. In addition, triethyl phosphate (TEP) and calcium salts are used to incorporate phosphate and calcium into the sol-gel system, respectively [10]. A gel is formed by polycondensation of the silica species in the sol, forming a network of silica (Si-O-Si bridging bonds) that subsequently goes through a heat treatment to remove the condensation by-products and remove the nitrates in cases where calcium nitrate salt is used, due to the cytotoxic nature of nitrate [6,9]. In addition, different drying techniques of wet gels result in porous structured materials known as aerogels and xerogels [9]. Aerogels are low-density gels that are produced by the removal of liquid from the interconnected pore network as a gas phase under supercritical drying; whereas xerogels, generically called gels, are the resulting product from drying at or near ambient conditions by thermal evaporation [9,11].

The advantage of sol-gel-derived over melt-quenching-derived BGs is the generation of different gel products, such as aerogels and gels that can be characterized for their applications as potential bioactive bone biomaterials [12,13]. In addition, the formation of an HCA layer on the surfaces of glasses and bondage to bone can be achieved with compositions of 90 mol% silica, whereas osteoconduction and osteoinduction property is achieved at a silica content of 60 mol% or less in melt-derived BGs [14]. The increased bioactivity of sol-gel-derived BGs is due to its enhanced surface area as a result of its nanoporous network, compared to the dense melt-derived BGs, causing an increased rate of dissolution of the soluble ionic species of the BG composition [15,16]. In addition, the incorporation of an organic component is more viable in the sol-gel process since it does not necessarily require high-temperature treatment, as would occur in the melt-derived BGs.

BGs are, however, brittle and require the incorporation of a polymer to induce toughness. The addition of biodegradable synthetic polymers, such as poly(ester amide) (PEA) [17], poly(caprolactone)(PCL) [2,18], poly(lactic-co-glycolic acid) (PLGA) [19], and polydimexylsiloxane (PDMS) [20], into BGs, have been extensively studied for bone tissue engineering [21]. Preparation of monolithic silica/polydimexylsiloxane/calcium phosphate composites (70%Si20%PDMS10%CaP and 60%Si20%PDMS20%CaP) have been prepared via the sol-gel process combined with a micro-molding technique at room temperature [20]. The bioactive properties of monolithic composites were assessed for their role as filling scaffolds in bone surgery with possible drug loading [20]. Results showed an increase in surface mineralization on 70%Si20%PDMS10%CaP composites which could potentially be used as a bone-filling material, whereas 60%Si20%PDMS20%CaP composites showed delayed initial mineralization with potential use as a drug-release material that bonds to bone [20].

Among the polymers that can be incorporated into the BG system, synthetic polymers offer increased mechanical strength compared to natural polymers. However, natural polymers are preferred because they possess macromolecules that the biological environment recognizes and metabolizes [22]. Hybrid xerogels composed of silica (SiO_2_)/chitosan (CS) and SiO_2_/CS/tricalcium phosphate (TCP) have been developed to evaluate the effects of washing treatments using either ethanol or water on the textural and bioactive properties for their potential application in bone regeneration [23]. Gels washed in ethanol resulted in an increased surface area, pore volume, and pore size compared to gels washed in water. In addition, xerogels containing TCP presented a higher ability to form hydroxyapatite on hybrids, promoting the adhesion of cells and proliferation of osteoblasts [23].

Selection of a natural polymer that better mimics the organic component of bone would, however, be beneficial for developing bone biomaterials. Due to the possible antigenic responses from collagen [24], gelatin, the hydrolyzed form of type I collagen, could be a suitable polymer. The application of gelatin to develop bone biomaterials is relatively cheap, readily available, and can easily be dispersed in aqueous solutions [25,26]. Gelatins are polymers of a mixture of amino acid moieties joined by peptide bonds ranging from 15,000 and 400,000 Da in molecular weight [27]. The primary structure of gelatin is composed of more than twenty amino acids in different proportions in such a way that their molecules are composed of repeating sequences of glycine-X-Y triplets, where X and Y describe the positions of proline and hydroxyproline, respectively [27]. Moreover, interactions between cells and the ECM are regulated by an arginine-glycine-aspartic acid (RGD) sequence present within the gelatin structure, which also functions as a specific integrin recognition site that promotes cell adhesion, preventing cells from apoptosis as well as accelerating tissue regeneration and therefore, functioning as a biomimetic peptide [27,28]. Furthermore, gelatin is able to molecularly interact with functional groups of organic and inorganic components that can be tailored to present specific physical properties necessary for the development of bioactive bone biomaterials. In the development of a tissue-engineered biomaterial for bone repair via a sol-gel process, gelatin has been used as a toughening polymer since BGs are brittle and cannot be implanted in mechanically stressed bone sites [29]. Gelatin by itself would also be too weak to support a bone scaffold. Therefore, the development of a nanocomposite material where BG and gelatin act as the inorganic and organic components, respectively, would potentially improve the bioactivity while serving as a bone scaffold template for the body to repair and regenerate itself [25]. Therefore, combining both gelatin and BG would create a nanocomposite that mimics the hierarchical organic–inorganic structure of bone.

The development of organic–inorganic nanocomposites should possess bioactive features that would enhance their applications as bone biomaterials. Calcium is fundamental to the bioactivity of sol-gel tertiary BGs and a key component of osteogenesis [30,31]. Calcium nitrate has been conventionally used as a calcium source to prepare BGs; however, its use has some disadvantages. The heterogeneity caused by calcium-rich regions and thermal treatments (>400 °C) to incorporate calcium ions into the silicate glass network are some of the drawbacks [32,33,34,35]. However, its major limitation comes from its incompatibility to incorporate a polymer component for the preparation of organic–inorganic nanocomposites at room temperature because of the high-temperature treatment needed to thermally decompose the nitrate [1,36].

Different calcium sources that can be used at lower temperatures are required for the synthesis of sol-gel-derived organic–inorganic nanocomposites. The use of calcium chloride (CaCl_2_), an alternative calcium salt, or calcium ethoxide (Ca(OEt)_2_), a calcium alkoxide, has been previously reported for the development of nanocomposite bone biomaterials [1,2,18]. A study has also investigated the effects of various calcium sources, such as calcium methoxyethoxide (CME), calcium nitrate and CaCl_2_, on the properties of sol-gel-derived two-component (SiO_2_-CaO)-based BGs and found that CME was a more suitable calcium source for its potential application in nanocomposite synthesis [30]. A separate study also evaluated the effects and properties of binary (SiO_2_-CaO) BGs composed of the above-mentioned calcium sources, including Ca(OEt)_2_, and assessed their biocompatibilities in a chitosan-BG composite model [37]. Their findings showed that Ca(OEt)_2_ was the preferred calcium source, showing higher calcium incorporation into the silicate network, homogeneity, bioactivity and biocompatibility [37]. Another study has additionally compared the incorporation of calcium ions from Ca(OEt)_2_, calcium hydroxide, CaCl_2_, calcium citrate and calcium acetate into the silicate network of binary BGs [31]. Calcium hydroxide was selected as an alternative to calcium alkoxides for the synthesis and characterizations of polycaprolactone-BG nanocomposite scaffolds synthesized at room temperature [31]. These studies have made possible the understanding of the role and function of calcium sources in the silicate BG network and their application as a biomaterial that mimics the composition of bone.

Furthermore, the addition of carbon-based conductive materials has also been incorporated into organic–inorganic compositions as a new generation of biomaterials that provide additional functionality for bone substitutes, namely conductivity [28,38]. Bone possesses natural conductive properties [39,40], and the addition of a conductive element into an organic–inorganic bone substitute could better mimic the natural electrical conductivity of bone, providing advantages at the physiological and mechanical levels [41,42]. Most importantly, carbon-based conductive materials could deliver electrical signals within a bone biomaterial through the application of electrical stimulation for the maturation of osteoblasts and to induce the repair and regeneration of bone defects [43,44]. Therefore, uniform distribution of a conductive component into organic–inorganic nanocomposites without affecting the overall properties of the synthesized bone biomaterials is beneficial for electrical stimulation future studies.

Herein, we report the effects of CaCl_2_ and Ca(OEt)_2_ as calcium sources to develop an efficient approach that incorporates gelatin, sol-gel-derived tertiary BG, and uniformly dispersed multiwall carbon nanotubes (MWCNTs) to create bone nanocomposites that mimic the organic–inorganic composition of bone with an electrically conductive element. Incorporation of distributed MWCNT into gelatin-BG nanocomposites was possible and could allow the study of potential future exploration of exogenous electrical stimulation on the maturation of osteoblasts. The main objective of this study was to compare the physicochemical properties of CaCl_2_- and Ca(OEt)_2_-based nanocomposites, as well as their elemental distribution within the gelatin-BG-MWCNT biomaterials. In addition, the swelling, degradation behavior and porosity of the nanocomposites were evaluated. Finally, we explored the in vitro bioactive properties of nanocomposites and their suitability to support cell attachment and spreading. Our data showed that gelatin–BG–MWCNT nanocomposites containing CaCl_2_ or Ca(OEt)_2_ possessed different physicochemical properties due to the fate of their calcium ions in the silicate glass network of the biomaterial. Although Ca(OEt)_2_-based nanocomposites were most likely to have more advantageous properties in bone-repairing applications, both CaCl_2_- and Ca(OEt)_2_-based gelatin–BG–MWCNT nanocomposites have the potential to function as bone-repair biomaterials.

## 2. Materials and Methods

### 2.1. Materials

Gelatin type A (porcine skin), Pluronic F-127, multiwall carbon nanotube (MWCNT, >98% carbon basis, O.D. × L 6–13 nm × 2.5–20 μm), tetraethyl orthosilicate (TEOS, 98%), triethyl phosphate (TEP, 99.8%), and anhydrous calcium chloride were purchased from Sigma–Aldrich (Milwaukee, WI, USA). Calcium ethoxide was obtained from Gelest Inc. (Morrisville, PA, USA). Dulbecco’s Modified Eagle’s Medium (DMEM), Hanks’ Balanced Salt Solution (HBSS), Fetal Bovine Serum (FBS), and penicillin/streptomycin (pen/strep) were acquired from Thermo Fisher (Whitby, ON, Canada). Alexa Fluor^®^ 488 phalloidin and 4′6-diamidino-2-phenylindole (DAPI) were purchased from Life Technologies (Burlington, ON, Canada). Mouse embryo multipotent mesenchymal progenitor cells (C3H/10T1/2 cells) were obtained from ATCC (Manassas, VA, USA).

### 2.2. Preparation of Sol-Gel Derived Gelatin-BG-MWCNT Nanocomposites Using Calcium Chloride and Calcium Ethoxide

To prepare gelatin–BG–MWCNT nanocomposites based on calcium chloride (CaCl_2_), 20 mg/mL of MWCNT was dispersed in Pluronic F-127 (PF-127) solution, which was previously dissolved in water at a high temperature to a concentration of 20 mg/mL, followed by sonication for 2 h at 50 °C and stored at RT until further use. Gelatin type A (porcine skin) was dissolved in water at a concentration of 10% *w*/*v*. Meanwhile, BG was prepared by sol-gel process from TEOS, TEP, and CaCl_2_ by adding water and a catalytic amount of 1M HCl for 30 min at room temperature. To prepare 100 mg of BG, 248 µL TEOS, 22 µL TEP and 49 mg of CaCl_2_ were used. The mole ratio of water/TEOS was kept at 4:1, while 1M HCl was added at a volume ratio (water/HCl) of 3 to catalyze the TEOS hydrolysis. Viscous gelatin solution was added dropwise to the tertiary glass precursors solution, followed by MWCNT (1 wt.%). Hydrolysis and polycondensation of TEOS were carried out in situ while the sol–gelatin–MWCNT mixture was vigorously stirred at 40 °C until it became a gel. BG had a final molar composition of 70% SiO_2_, 26% CaCl_2_, and 4% P_2_O_5,_ and the organic–inorganic ratio was maintained at 50 wt.%. Aging of gels was carried out at 55 °C for one day, followed by drying under vacuum for one day at 60 °C. The final product was then ground to a fine powder. Table 1 presents the nomenclature of the samples.

To prepare gelatin–BG–MWCNT nanocomposites based on calcium ethoxide (Ca(OEt)_2_), PF-127 was used to prepare MWCNT stock dispersions (20 mg/mL PF-127 and 20 mg/mL MWCNT). Dispersions were sonicated for 1 h at 50 °C and stored at RT until further use. Gelatin type A (porcine skin) was dissolved in water at a concentration of 10% *w*/*v*. A total of 1 wt.% MWCNT was added to the gelatin solution, followed by sonication at 50 °C for 30 min. BG (100 mg) was prepared by a sol-gel process [1,45], which consisted of hydrolyzing TEOS (248 µL) and TEP (22 µL) with a catalytic amount of 1M HCl under vigorous stirring at RT. Calcium ethoxide was dissolved separately in 2-ethoxyethanol (54 mg/mL). The hydrolyzed BG precursors were added dropwise to the gelatin–MWCNT mix, followed by calcium ethoxide. BG had a final molar composition of 70% SiO_2_, 26% CaO, and 4% P_2_O_5,_ and the organic–inorganic ratio was maintained at 50 wt.%. Biomaterials were aged for one day at RT, followed by drying under vacuum at 40 °C. Fine powder was obtained from the final product by grinding with a pestle and mortar. Sample nomenclatures are presented in Table 1.

### 2.3. Characterization of Nanocomposites

#### 2.3.1. Attenuated Total Reflectance Fourier Transform Infrared Spectroscopy (ATR-FTIR)

Dried and powdered nanocomposites were used for FTIR spectroscopy, which was conducted using a Thermo Scientific™ Nicolet™ Summit FTIR Spectrometer with the Everest ATR Accessory in the transmission mode at a resolution of 4 cm^−1^ and sample scans of 24 in the range of 4000–500 cm^−1^.

#### 2.3.2. Scanning Electron Microscopy (SEM) and Energy Dispersive X-ray Spectroscopy (EDX) Analysis

Nanocomposite disks were prepared to visualize the surface morphology and elemental distribution of their chemical composition through SEM and EDX, respectively. Briefly, 70 mg of dried gelatin–BG–MWCNT were compress-molded at 121 °C with a pressure of 145 psi for 50 min to obtain nanocomposite disk samples of 6 mm diameter and 1.5 mm height. Disks were sputter-coated with gold/palladium (K550X, sputter coater, Emitech Ltd., Ashford, UK), and SEM coupled to EDX was performed by using a Zeiss 1540XB FIB/SEM instrument with an accelerating voltage of 5 kV (Carl Zeiss: Oberkochen, Germany).

#### 2.3.3. Swelling Behavior of CaCl_2_- and Ca(OEt)_2_-Based Nanocomposites

Evaluation of the swelling behavior of nanocomposite disks (*n* = 3) was performed in PBS for 6 days at 37 °C. The weights of the samples were recorded before (*W*_0_) and after incubation in PBS after 1, 3, and 6 days of incubation (*W_t_*). The swelling ratio of samples was calculated according to Equation (1):

(1) $$Swelling\;Ratio\;\left(\%\right)=\frac{W_{t}-W_{0}}{W_{0}}\;\times 100$$

#### 2.3.4. In Vitro Biodegradability of CaCl_2_- and Ca(OEt)_2_-Based Nanocomposites

The weight loss percentages of nanocomposite disks (*n* = 3) were determined by measuring the initial weight of the samples (*W*_0_) and subsequently incubating in PBS at 37 °C. At 1, 3, and 6 days of incubation, the samples were washed with deionized water and dried under vacuum at RT. The final weights of the dried samples were recorded (*W_f_*) and were used to calculate the weight loss percentages of each sample, as presented in Equation (2).

(2) $$Weight\;loss\;\left(\%\right)=\frac{W_{0}-W_{f}}{W_{0}}\;\times \;100$$

#### 2.3.5. Micro-CT imaging of CaCl_2_- and Ca(OEt)_2_-Based Nanocomposites Post-Degradation

The morphology of nanocomposite disks after 6 days of degradation was imaged and studied using microcomputed tomography (microCT) (eXplore Locus SP, GE Healthcare, London, ON, Canada). Nanocomposite disks were scanned at 20 μm voxel resolution, using an exposure time of 0.45 s, 5 frames per view, and a total of 900 views at an increment of 0.4°. Two-dimensional slice images were reassembled from the isotropic slice data and compiled to generate a 3D image. 3D images were analyzed using commercially available trabecular bone analysis software MicroView version 2.5.0-4196 (GE Healthcare Biosciences). The threshold values distinguishing the nanocomposites from air were selected by using air and water as control objects. Analyses of micro-CT images include porosity measurements, pore wall thickness, pore sizes, and surface area to volume ratio of the nanocomposites.

#### 2.3.6. In Vitro Bioactivity of CaCl_2_- and Ca(OEt)_2_-Based Nanocomposites

Nanocomposite disks were incubated in simulated body fluid (SBF) solution at a concentration of 10 mg/mL at 37 °C under constant shaking at 120 rpm for 7 days. After SBF incubation, nanocomposites were rinsed with water, dried under vacuum at RT, and their surfaces were visualized by SEM/EDX using Zeiss 1540XB FIB/SEM instrument with an accelerating voltage of 5 kV (Carl Zeiss: Oberkochen, Germany). FTIR and X-ray diffraction (XRD) data were acquired on dried samples. XRD data were obtained using an X-ray diffractometer Rigaku Ultima III operating on Cu Kα radiation with λ = 1.5418 Å at 30 kV and 15 mA in the 2θ range of 2–90° at a scanning speed of 2°/min and scanning width of 0.02°.

#### 2.3.7. Cell Adhesion and Viability of CaCl_2_- and Ca(OEt)_2_-Based Nanocomposites

10T1/2 cells were used to investigate cell adhesion onto nanocomposites. Nanocomposite disks were disinfected under ultraviolet (UV) light and pretreated in HBSS (~10–15 min) to pre-wet the samples [5]. 10T1/2 cells (cultured in DMEM with 5% FBS and 1% pen/strep) were subsequently directly seeded onto nanocomposite disks that were placed in a 24-well plate. The nanocomposite disks have a surface area of 0.3 cm^2^, thus the seeded cell density on nanocomposites was 98,956 cell/cm^2^ and were incubated at 37 °C in 5% CO_2_. After 24 h incubation, cells were fixed using 4% paraformaldehyde (EMD Chemicals Inc. Gibbstown, NJ, USA) and stained against DAPI (300 nmol in PBS) and phalloidin (1:100) to visualize cell nuclei and F-actin, respectively. Live/dead cell staining kit was used for 10T1/2 cells viability on standard media and conditioned media (extracts) after 24, 72, and 168 h according to the manufacturer’s protocol. Conditioned media was prepared by incubating nanocomposites in fresh DMEM (5% FBS, 1% pen/strep) for 24 h at 37 °C. In a typical experiment, ~70 mg nanocomposite disks (6 mm diameter and 1.5 mm height) were incubated in 7 mL DMEM, corresponding to 10 mg/mL. After 24 h, the media which were conditioned with nanocomposite extracts was used as cell culture media to perform viability studies in a 24-well plate (working volume was 1 mL conditioned media). The same number of cells that were plated onto nanocomposites were evenly plated into a 24-well plate, resulting in a cell density of 15,625 cell/cm^2^. Standard culture media and conditioned media were changed every other day. Cells seeded on a tissue culture plate (TCP) were used as control. Images were taken with a Leica DMi8 fluorescence microscope (Leica Microsystems CMS GmbH, Wetzlar, Germany). Experiments were done in triplicate.

#### 2.3.8. Statistical Analysis

Statistical analysis of the data was performed using GraphPad Prism (https://www.graphpad.com/). Differences were tested with one-way ANOVA, and a *p*-value of <0.05 was used for statistical significance.

## 3. Results and Discussion

### 3.1. Preparation of Gelatin-BG-MWCNT Nanocomposites from CaCl_2_- and Ca(OEt)_2_ Calcium Sources

Calcium is an important element in osseous tissue, and its presence in a nanocomposite biomaterial could mimic bone’s natural inorganic composition. Evaluation of the effects of calcium sources on the synthesized tertiary BG could provide a better understanding of their role in the glass network and their function within nanocomposite bone biomaterials.

**Figure 1:**
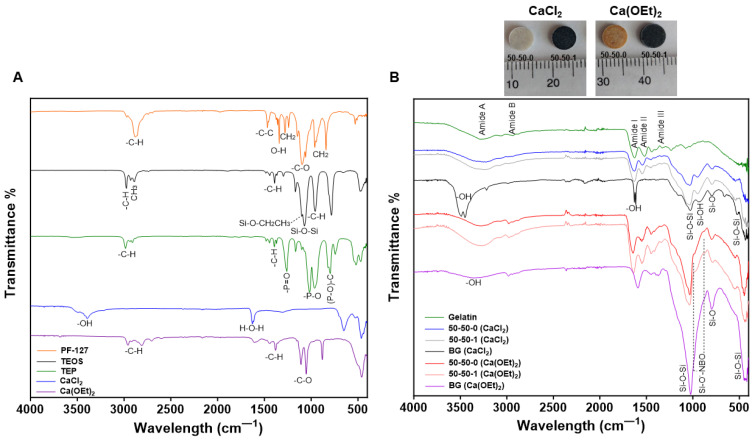
Chemical characterization of PF-127 surfactant, sol-gel precursors, organic, inorganic and nanocomposites using CaCl_2_ and Ca(OEt)_2_ as calcium sources. (**A**) FTIR spectra of PF-127 surfactant, sol-gel TEOS and TEP precursors, as well as CaCl_2_ and Ca(OEt)_2_ calcium sources. (**B**) Digital images of CaCl_2_- and Ca(OEt)_2_-based 50-50-0 and 50-50-1 nanocomposite disks. FTIR spectra of gelatin, BG containing CaCl_2_ and Ca(OEt)_2_, 50-50-0 and 50-50-1 nanocomposites composed of either CaCl_2_ and Ca(OEt)_2_.

Figure 1 presents the FTIR spectra showing the different chemical groups of the PF-127 surfactant, sol-gel precursors (Figure 1A) as well as of gelatin, tertiary BG using calcium chloride (CaCl_2_) or calcium ethoxide (Ca(OEt)_2_) as calcium sources, and their corresponding organic–inorganic nanocomposites with their corresponding digital images (Figure 1B). The bands associated with the PF–127 spectrum are characterized by bands at 2877 cm^−1^ corresponding to the –C–H (aliphatic region), 1473 cm^−1^ related to the C–C stretching, 1341 cm^−1^ associated with the O–H plane and 1101 cm^−1^ (region C–O) with three specific spots at 1061, 1106 and 1145 cm^−1^ that also suggest C–O–C stretching [46]. Peaks at 1286 cm^−1^ and 1287 cm^−1^ correspond to CH_2_ twisting, whereas bands at 956 cm^−1^ and 840 cm^−1^ are related to CH_2_ wobbling [46]. The sol-gel precursors used were TEOS and TEP, and the calcium sources were CaCl_2_ and Ca(OEt)_2_ (Figure 1A). TEOS, which was used as the silica precursor in the sol-gel process, possessed characteristics peaks at 2979, 2887, 1394, 1104, 1078 and 956 cm^−1^. The band at around 2979 cm^−1^ is assigned to the stretching vibrations of –C–H bond, while the band observed at 2887 cm^−1^ corresponds to the absorption band of the methyl (CH_3_) groups [47]. In addition, the peak at 1394 cm^−1^ is attributed to the asymmetric bending of –C–H bonds [48], while the band presented at 1104 cm^−1,^ corresponding to the ethoxy group bound to the silicon atom (Si–OCH_2_CH_3_), was observed. The peak at 1078 cm^−1^ is associated with the siloxane bonds (Si–O–Si), whereas the band at 956 cm^−1^ is characteristic of the –C–H rocking vibrations of TEOS [48,49]. Furthermore, the P_2_O_5_ precursor, TEP, also presented bands related to the symmetric stretching vibrations of –C–H bonds at 2987 and 2907 cm^−1^, as well as peaks related to the asymmetric bending of the –C–H bonds at around 1500 and 1368 cm^−1^ [50]. The prominent peak observed at 1018 and 965 cm^−1^ corresponds to the –P–O valence vibrations, while the band at 1263 cm^−1^ is attributed to the –P=O stretching vibrations of TEP [50]. Moreover, CaCl_2_ presented peaks observed at 3503 and 3393 cm^−1^ associated with the asymmetric –OH stretch, whereas the band at 1629 cm^−1^ corresponded to the H–O–H bending vibration of CaCl_2_ [51,52]. Although we have used anhydrous calcium chloride, its hygroscopic nature during sample transfer is the likely source of the –OH stretch. The appearance of the bands corresponding to –OH and H–O–H indicates the existence of different hydrogen bonding environments resulting from interactions with chloride ions as well as water molecules [51]. Additional peaks at around 650 cm^−1^ are associated with the Ca–O stretching vibration [53]. Lastly, Ca(OEt)_2_ presented peaks between 3000 and 2800 cm^−1^ due to the C–H stretching, as well as an alkane (–C–H) bending at 1400 cm^−1^ [54]. In addition, the bands observed between 1100 and 1050 cm^−1^ are characteristic of the primary alcohol (–C–O) stretching [54].

The tertiary BG containing CaCl_2_ presented characteristic peaks between 3496 and 3454 cm^−1^ due to the –OH stretching and bending vibrations of the self-associated silanol (Si–OH) groups (Figure 1B). In the Ca(OEt)_2_-based tertiary BG, this peak is shifted slightly to 3370 cm^−1^. However, the CaCl_2_-based BG also presented an additional peak at 1630 cm^−1^ due to the self-associated Si–OH groups [45] which overlapped with the peak associated with the residual water of CaCl_2_ [51,52]. In the BG containing Ca(OEt)_2_, there were bands associated with C–H vibrational modes at around 2900 and 1400 cm^−1^. While incomplete hydrolysis could be a suspect for these peaks, this cannot explain it fully, especially since we repeated the experiment with longer hydrolysis and drying times (96 h). Interestingly, the peak at around 2900 cm^−1^ has been observed even in a melt-derived CaO-SiO_2_ glass with no obvious source of CH_2_ groups and persisted even after SBF treatment [55]. We also observed this small peak in our previous study of the SiO_2_-P_2_O_5_ system after 72 h hydrolysis and 7 days of aging [45]. Therefore, the data indicate that these peaks are not necessarily associated with incomplete hydrolysis of the inorganic components. Furthermore, we observed peaks at 1025 cm^−1^ due to the asymmetric stretching of the siloxane bond (Si-O-Si) as well as a band at around 800 cm^−1^ associated with the bending Si-O vibration of the ring structures of the glass for both CaCl_2_- and Ca(OEt)_2_-based BGs [56] (Figure 1B). Both CaCl_2_- and Ca(OEt)_2_-synthesized BGs presented a band at around 450 cm^−1^ corresponding to the rocking motion of oxygen bridging two adjacent silica atoms from the Si-O-Si groups of the glass network [1]. BG containing CaCl_2_ as a calcium source presented a peak at 943 cm^−1^ related to the silanol (Si–OH) groups from the incomplete polycondensation of the TEOS (Figure 1B) [45]. In contrast, evidence of peaks showing the incorporation of calcium ions to the silicate glass network was observed in the Ca(OEt)_2_-based BG as shown by the peaks at 984 cm^−1^ and 887 cm^−1^, corresponding to the Si-O^−^-non-bridging oxygen (NBO) (Figure 1B) [57]. Evidence showing the incorporation of calcium ions to the silicate glass network was observed by the broader Si-O-Si bands in the Ca(OEt)_2_-based BG. The broader bands indicate increased disorganization and decreased polymerization within the silicate glass network due to the incorporation of network-modifying calcium ions [37].

Furthermore, gelatin showed distinguishing bands at 3270 cm^−1^ and 2946 cm^−1^ associated with amide A and amide B, respectively. These bands corresponded to the stretching vibrations of the free N–H and O–H groups and the asymmetric stretching vibrations of =C–H and –NH_3_^+^ of the peptide fragments, respectively. Peaks corresponding to amide I at 1627 cm^−1^ as a result of the C=O stretching vibrations along the polypeptide backbone of gelatin was observed, as well as the presence of amide II at 1524 cm^−1^ corresponding to the N–H bending and C–N stretching vibrations [57,58]. An additional band at 1234 cm^−1^ was observed, indicating the presence of amide III which is associated with the wagging vibrations of CH_2_ groups from the glycine backbone and proline side chains as well as the presence of the vibration of stretching C–N bonds and the vibration of bending N–H bonds [59,60,61].

Gelatin-BG-MWCNT (50-50-1) nanocomposites composed of CaCl_2_ and Ca(OEt)_2_ showed peaks attributed to both the inorganic sol-gel-derived silica phase as well as the organic gelatin component (Figure 1B). In addition, Figure 1B shows the FTIR spectra of the 50-50-0 nanocomposites where the same peaks corresponding to the organic and inorganic elements of the 50-50-1 biomaterials, are observed. Therefore, no chemical shift is expected in the 50-50-0 nanocomposites. The presence of both organic–inorganic groups within the nanocomposites shows the existence of a physical bond between these elements within the biomaterial. In addition, the band corresponding to amide III for both CaCl_2_- and Ca(OEt)_2_-based 50-50-0 and 50-50-1 nanocomposites was decreased (Figure 1B). This characteristic feature has been observed before in another study and was reported to be due to the interaction between the silicon hydroxyl (Si–OH) groups of BG and the amino (–NH_2_) groups of gelatin polymer by electrostatic and hydrogen bonding [61]. Finally, calcium ions from the Ca(OEt)_2_-based 50-50-0 and 50-50-1 nanocomposites were also incorporated into the silicate glass network. This was observed from the peaks attributed to the NBO to form Si–O–Ca and the broader Si-O-Si bands in the Ca(OEt)_2_-based nanocomposites [37].

### 3.2. Surface Morphological and Elemental Distribution of 50-50-1 Nanocomposites Prepared from CaCl_2_- and Ca(OEt)_2_ Sources

**Figure 2:**
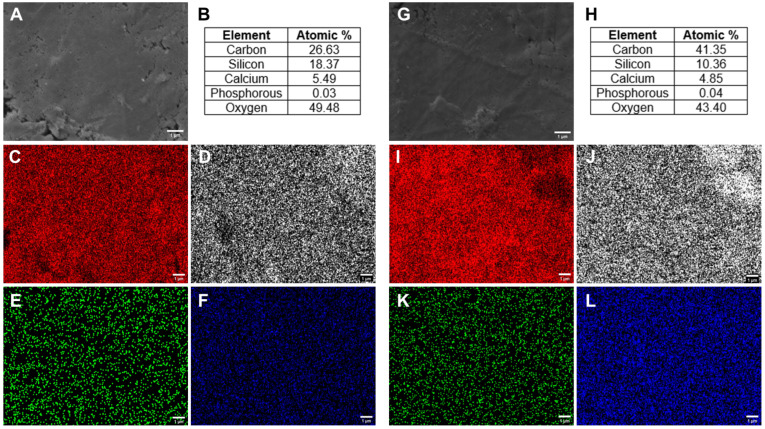
Surface elemental homogeneity in 50-50-1 nanocomposites using CaCl_2_ and Ca(OEt)_2_ as calcium sources. (**A**) SEM image, (**B**) atomic percentages of elements, and elemental mapping of (**C**) carbon, (**D**) silicon, (**E**) calcium, (**F**) phosphorous for the CaCl_2_-based 50-50-1 nanocomposite. (**G**) SEM image, (**H**) atomic percentages of elements, and elemental mapping of (**I**) carbon, (**J**) silicon, (**K**) calcium, (**L**) phosphorus for the Ca(OEt)_2_-based 50-50-1 nanocomposite. Scale bar = 1 µm.

Adequate dispersion of the elements of the nanocomposites is beneficial to ensure homogeneous incorporation of all components in the organic–inorganic system. Calcium is fundamental to the bioactivity of sol-gel BGs and a key component of osteogenesis [30,31]. The surface morphology and elemental distribution of nanocomposites using CaCl_2_ (Figure 2A–F) and Ca(OEt)_2_ (Figure 2G–L) as calcium sources for the preparation of 50-50-1 nanocomposites were visualized using SEM and EDX. The tables in Figure 2B,H show the atomic percentages of the elements in the specific region where the SEM was obtained. Although there is no elemental mapping for oxygen, its presence is characteristic due to the oxides in the tertiary BG (SiO_2_, CaCl_2_, P_2_O_5_ and SiO_2_, CaO, P_2_O_5_). Both CaCl_2_ and Ca(OEt)_2_-based 50-50-1 nanocomposites showed uniform elemental distribution of their organic and inorganic components. Carbon atoms were uniformly dispersed within the nanocomposite, representing the presence of gelatin and MWCNT (Figure 2C,I), whereas silicon, calcium, and phosphorous elements were attributed to the tertiary BG (Figure 2D–F,J–L).

Chemically, CaCl_2_ and Ca(OEt)_2_-based 50-50-1 nanocomposites are different since the calcium ions from the Ca(OEt)_2_ calcium source are incorporated within the nanocomposite network. Although evidence has shown that calcium chloride salts fail to enter the network even after applying any elevated temperatures [30], both CaCl_2_ and Ca(OEt)_2_-based 50-50-1 nanocomposites showed uniform elemental distribution. The advantages of using CaCl_2_ and Ca(OEt)_2_ as calcium sources for the preparation of in situ sol-gel organic–inorganic nanocomposites are that they are synthesized at room temperature, and good surface homogeneity of their elements was obtained.

### 3.3. Swelling Behavior of Nanocomposites Composed of CaCl_2_- and Ca(OEt)_2_ Calcium Sources

**Figure 3:**
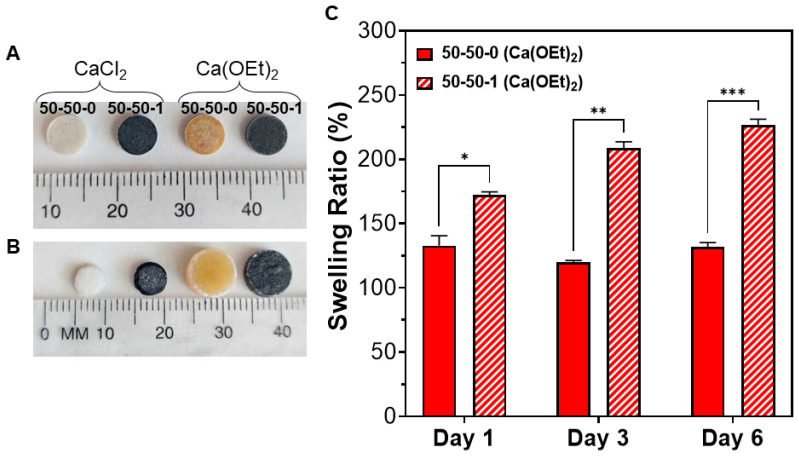
Swelling behavior of 50-50-0 and 50-50-1 nanocomposites composed of CaCl_2_ and Ca(OEt)_2_ calcium sources. Digital images of nanocomposites as-prepared in dry state (**A**) and after (**B**) incubation in PBS for 6 days in wet state. (**C**) Swelling ratio of Ca(OEt)_2_-based nanocomposites throughout 6 days of incubation. * *p* < 0.05, ** *p* < 0.01, *** *p* < 0.001.

The swelling of nanocomposites is an important property that ensures nutrient transport and removal of waste products for their application as bone biomaterials. Following the observation that CaCl_2_ and Ca(OEt)_2_-based nanocomposites are chemically different but show homogeneous incorporation of the organic–inorganic element system, an assessment of the swelling behavior as a function of calcium sources was conducted. Digital images of the swelling behavior of nanocomposite disks are shown before and after incubation in PBS for 6 days (Figure 3A,B). The digital images demonstrate that Ca(OEt)_2_-based nanocomposites swell and their diameters increased to 9 mm. However, CaCl_2_-based nanocomposites did not swell after incubation in PBS; instead, their weight was reduced, and the diameter of the disks decreased by 1 mm. Therefore, the swelling ratio of the CaCl_2_-based nanocomposites was not plotted in Figure 3C since the water uptake of the biomaterials was not applicable. However, for Ca(OEt)_2_-containing nanocomposites, swelling ratios of 130% (composition 50-50-0) and 226% (composition 50-50-1) were observed after 6 days of incubation. The increasing swelling behavior observed in the 50-50-1 nanocomposite could be due to the addition of MWCNTs to the organic–inorganic system, leading to a higher surface area and increasing its water uptake [62,63].

The difference in swelling behavior is explained by the sources of calcium used to prepare the nanocomposites and the fate of gelatin in the system. Calcium ions in the Ca(OEt)_2_-based nanocomposites are incorporated into the matrix, therefore entrapping the gelatin into the nanocomposite network, which causes swelling. In addition, the presence of strong ionic interactions between the released calcium ions from the BG with the carboxylic groups in gelatin causes aggregation of the polymer chains within the nanocomposite system [64,65]. The calcium in CaCl_2_-containing nanocomposites, however, are not bonded in the matrix which results in mass loss due to the diffusion of calcium salts. In addition, the existence of weaker interactions between the organic and inorganic components causes the gelatin to leach out faster and, therefore, limits the swelling of the nanocomposite. Thus, Ca(OEt)_2_-based nanocomposites, especially the 50-50-1, could function better as a bone biomaterial and could likely lead to efficient nutrient transport and removal of waste products for its application for the repair and regeneration of bone.

### 3.4. In Vitro Biodegradation Study of CaCl_2_- and Ca(OEt)_2_-Based Nanocomposites

**Figure 4:**
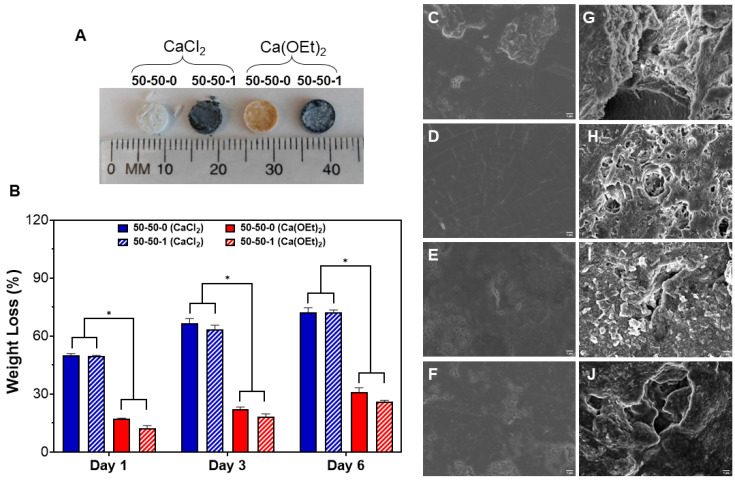
In vitro biodegradation study of 50-50-0 and 50-50-1 nanocomposites composed of CaCl_2_ and Ca(OEt)_2_ calcium sources. (**A**) Digital images of CaCl_2_- and Ca(OEt)_2_-based 50-50-0 and 50-50-1 nanocomposites after 6 days of degradation. (**B**) Biodegradation behavior of CaCl_2_- and Ca(OEt)_2_-based 50-50-0 and 50-50-1 nanocomposites within 6 days. SEM images of nanocomposites before (**C**) 50-50-0 (CaCl_2_), (**D**) 50-50-1 (CaCl_2_), (**E**) 50-50-0 (Ca(OEt)_2_), (**F**) 50-50-1 (Ca(OEt)_2_) and after (**G**) 50-50-0 (CaCl_2_), (**H**) 50-50-1 (CaCl_2_), (**I**) 50-50-0 (Ca(OEt)_2_), (**J**) 50-50-1 (Ca(OEt)_2_) 6 days of degradation. Scale bar = 1 µm. * *p* < 0.05.

The degradation behavior of 50-50-0 and 50-50-1 nanocomposites containing either CaCl_2_ or Ca(OEt)_2_ was performed for 6 days in PBS. Digital images were taken of the nanocomposite disks before (Figure 3A) and after (Figure 4A) degradation. Nanocomposites containing CaCl_2_ as a calcium source were very brittle, as observed by the fragmented pieces after drying. In contrast, nanocomposites containing Ca(OEt)_2_ as the calcium source were dimensionally stable (Figure 4A).

The weight loss of 50-50-0 and 50-50-1 nanocomposites containing either CaCl_2_ or Ca(OEt)_2_ are shown in Figure 4B. Weight loss percentages of CaCl_2_-based 50-50-0 and 50-50-1 nanocomposites were 50% after one day of degradation. However, Ca(OEt)_2_-based nanocomposites showed significantly less weight loss percentages of 17% and 12% for the 50-50-0 and 50-50-1 nanocomposites, respectively. After 3 days of degradation, the 50-50-0 and 50-50-1 nanocomposites containing CaCl_2_ continued degrading and presented weight loss percentages of 66% and 63%, respectively. In contrast, Ca(OEt)_2_-based 50-50-0 and 50-50-1 nanocomposites showed a significantly decreased degradation of only 22% and 18%, respectively. This tendency was observed throughout the entire degradation period where at day 6 of degradation, CaCl_2_-based nanocomposites showed a weight loss percent of 72%, but was low for the Ca(OEt)_2_-containing biomaterials which were of 30% and 26% for the 50-50-0 and 50-50-1 nanocomposites, respectively. The significant difference in weight loss is due to the calcium sources used to prepare the sol-gel BG. The considerable mass loss of nanocomposites produced with CaCl_2_ was due to the unsuccessful incorporation of the calcium ions derived from the chloride salt to enter the silicate network [37]. However, the Ca(OEt)_2_ calcium source was involved in the in situ inorganic polymerization of the sol-gel BG during nanocomposite synthesis. This resulted in the incorporation of calcium ions into the silicate network [30,66], as confirmed in Figure 1B, causing a decreased weight loss percentage. As stated in the Section 2, we used 0.234 g (0.001125 mol) TEOS, 0.024 g (0.000132 mol) TEP, and 0.049 g (0.000441 mol) CaCl_2_ or 0.0541 g (0.000416 mol) Ca(OEt)_2_ to synthesize tertiary BG. After the stoichiometric reaction, 0.001125 mol of SiO_2_, 0.0000659 mol P_2_O_5_, and 0.000441 mol CaCl_2_ or 0.000416 mol CaO will be produced. These correspond to a molar composition of ~70% SiO_2_, ~4% P_2_O_5_, and ~26% CaCl_2_ or CaO. Thus, the mass loss data for the CaCl_2_-based system indicated that both gelatin and CaCl_2_ were lost (the sum of gelatin + CaCl_2_ in the formulation was ~69.5% on the basis of mass). Our results would therefore indicate that Ca(OEt)_2_-based nanocomposites could become a better calcium source for synthesizing organic–inorganic bone biomaterials.

The morphology of the nanocomposites before and after degradation was observed. Figure 4C–F show the SEM images of the surfaces of 50-50-0 (Figure 4C,E) and 50-50-1 (Figure 4D,F) before degradation where the calcium source used was either CaCl_2_ (Figure 4C,D) or Ca(OEt)_2_ (Figure 4E,F). A non-porous structure with some roughness due to the compression molding process of the biomaterials were observed for all nanocomposites. However, nanocomposites presented a porous structure after the degradation period (Figure 4G–J). Formation of a porous structure post-degradation was most evident in nanocomposites containing CaCl_2_ (Figure 4G,H) than Ca(OEt)_2_ (Figure 4I,J). Degradation of BG occurs through hydrolysis of the Si-O-Si bonds of the glass structure, forming Si(OH)_4_ and silanol. The presence of calcium, as a network modifier, accelerates the degradation process since it disrupts the silicate network. This is the case for nanocomposites composed of Ca(OEt)_2_. However, in the case of CaCl_2_-based BG, the calcium ions are therefore entrapped physically in the glass network and can diffuse out easily upon contact with water or biological fluids. Pure BGs are inherently brittle regardless of the calcium sources that are being used, but toughness is conferred upon the addition of gelatin [29]. Degradation of gelatin occurs hydrolytically and enzymatically [67] and possesses a melting temperature of ~30 °C. The gelatin component of the synthesized gelatin–BG–MWCNT nanocomposite would be leaching out of the biomaterial, creating a porous structure. For bone-repair applications, the interconnected porous structure could potentially allow cells to infiltrate the pores of the nanocomposites and lay down their extracellular matrix (ECM) to remodel and regenerate bone.

### 3.5. Microstructure, Pore Size and Porosity of CaCl_2_- and Ca(OEt)_2_-Based Nanocomposites

**Figure 5:**
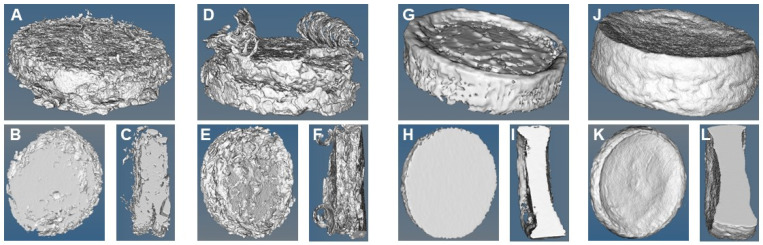
Micro-CT images of CaCl_2_- and Ca(OEt)_2_-containing 50-50-0 and 50-50-1 nanocomposites after 6 days of degradation. (**A**–**C**) 50-50-0 (CaCl_2_), (**D**–**F**) 50-50-1 (CaCl_2_), (**G**–**I**) 50-50-0 (Ca(OEt)_2_), (**J**–**L**) 50-50-1 Ca(OEt)_2_). (**B**,**E**,**H**,**K**) are horizontal cross-sections of nanocomposites. (**C**,**F**,**I**,**L**) are vertical cross-sections of nanocomposites.

Micro-CT imaging was obtained to better visualize the morphology and investigate the porosity, pore wall thickness and pore size distribution of the nanocomposites after 6 days of degradation. Figure 5 shows the micro-CT isosurface images of 50-50-0 and 50-50-1 nanocomposites containing either CaCl_2_ or Ca(OEt)_2_ calcium sources. The CaCl_2_-based nanocomposites depict a more porous and brittle structure, where the biomaterial appears to have been disintegrating (lifted) as a result of the increased degradation. In contrast, Ca(OEt)_2_-based nanocomposites have a more compact and tougher structure. The organic–inorganic nanocomposites post-degradation present reduced pore sizes and a decreased porous structure because the gelatin component was added as the polymer matrix and leached out throughout the whole nanocomposite.

The average porosity of the 50-50-0 and 50-50-1 nanocomposites composed of CaCl_2_ were 24.48% and 29.59%, respectively, whereas for the 50-50-0 and 50-50-1 nanocomposites composed of Ca(OEt)_2_ were 5.03% and 5.84% (Table 2). The calcium in nanocomposites containing CaCl_2_ was expected to leach out since the calcium ions are not bonded in the nanocomposite matrix, therefore resulting in more porosity after degradation. However, the low porosity found in Ca(OEt)_2_-based nanocomposites would most likely come from the gelatin that had been leached during degradation since the calcium ions form part of the organic–inorganic matrix. Furthermore, pore wall thickness increased with the Ca(OEt)_2_-based nanocomposites. 50-50-0 and 50-50-1 CaCl_2_-based nanocomposites presented pore wall thickness values of 0.49 µm and 0.41 µm, respectively, while the values for Ca(OEt)_2_-based nanocomposites were 1.64 µm and 1.92 µm. In addition, the pore sizes of nanocomposites containing CaCl_2_ were 0.16 µm for both 50-50-0 and 50-50-1 nanocomposites. However, decreased pore size values were obtained for the 50-50-0 and 50-50-1 Ca(OEt)_2_-containing nanocomposites which were 0.06 µm and 0.10 µm, respectively. Although the pore sizes of the nanocomposites are not large enough to allow cell infiltration into the porous structure, the generation of micropores (0.1-10 µm pore size) improves cell attachment by creating a rough surface allowing penetration of body fluids [68,69]. In addition, the generation of nanopores (<0.10 µm) creates a larger surface area that increases the bioactive properties of the materials and stimulates greater ion exchange and protein adsorption, which are favorable for bone repair applications [69,70,71]. The surface area to volume ratio was higher for CaCl_2_-based nanocomposites, where the values for 50-50-0 and 50-50-1 nanocomposites were 4.08 mm^−1^ and 4.90 mm^−1^, respectively, while the values for Ca(OEt)_2_-based nanocomposites were 1.22 mm^−1^ and 1.04 mm^−1^, respectively.

### 3.6. In Vitro Bioactivity of CaCl_2_- and Ca(OEt)_2_-Based 50-50-0 and 50-50-1 Nanocomposites

**Figure 6:**
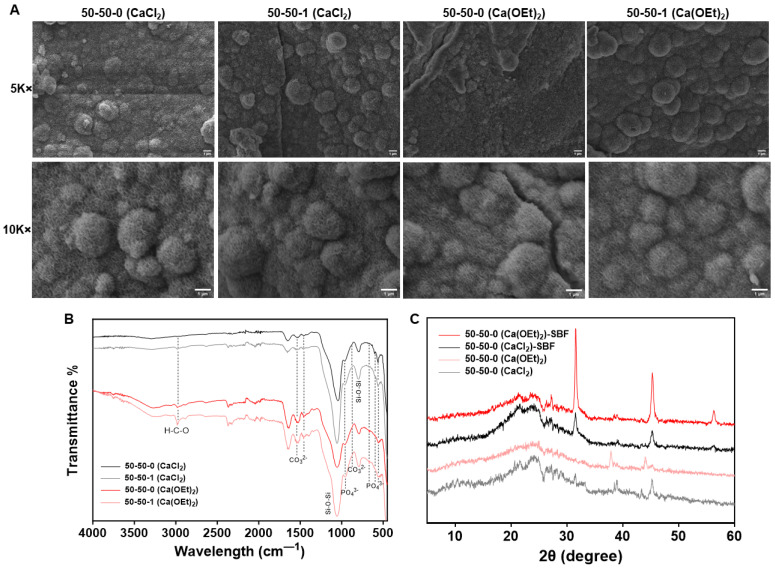
In vitro bioactivity of CaCl_2_- and Ca(OEt)_2_-based 50-50-0 and 50-50-1 nanocomposites. (**A**) SEM images of the surfaces of nanocomposites after SBF incubation for 7 days. Scale bar = 1 µm. (**B**) FTIR and (**C**) XRD spectra of CaCl_2_- and Ca(OEt)_2_-based 50-50-0 and 50-50-1 nanocomposites after SBF treatment for 7 days.

Bone substitutes should be capable of possessing bioactive properties to promote the formation of bone. Due to the nature of BG, its presence within the organic–inorganic nanocomposites should provide bioactive features that induce the formation of hydroxycarbonate apatite (HCA) (Ca_10−x_(PO_4_)_6−x_(CO_3_)_x_(OH)_2−x_) which is normally found in bone. The formation of HCA layers occurs through the release of soluble ionic species from the tertiary BG component, such as Si, Ca^2+^, and PO_4_^3−^ ions, into the simulated body fluid (SBF) solution. The release of these ions is followed by their re-deposition to form a high-surface-area hydrated silica and polycrystalline HCA bilayer on the glass surface, similar to that of bone [18,28]. Evaluation of the HCA formation on the surfaces of the organic–inorganic nanocomposite disks was performed by incubating in SBF. Figure 6A shows the SEM images of nanocomposites after incubation in SBF for 7 days. Although the formation of HCA can be evaluated at different time points ranging from day 0 to day 10 [18,72], day 7 represents a time point in which the HCA formation is comparable to that of further treatment periods in SBF. Regardless of the calcium sources used for the synthesis of the inorganic component within the organic–inorganic nanocomposite, characteristic spherical-shaped HCA crystallized particles were observed on the surfaces of 50-50-0 and 50-50-1 nanocomposite disks after incubation in SBF for 7 days. Table 3 presents the atomic percentages of Ca and P before and after incubation in SBF as well as the Ca/P ratio of CaCl_2_- and Ca(OEt)_2_-based nanocomposites after SBF treatment. The results show that the nanocomposites were enriched with Ca and P, the main components of HCA in bone, as observed in the increased atomic percentages of Ca and P on the surfaces of nanocomposites after SBF incubation (Table 3). Additionally, the resulting Ca/P ratios after SBF treatment were higher than the stoichiometric Ca/P ratio of hydroxyapatite (Ca_10_(PO_4_)_6_(OH)_2_), a typical artificial bone [73], which is between 1.67 and 1.50. The increased Ca/P ratio in the nanocomposite could be due to the carbonate ion substitution in the HCA structure [74]. In addition, the incorporation of 1 wt.% MWCNT did not interfere with the formation of the HCA layer on the surfaces of nanocomposites. The increased atomic percentages of Ca and P in the Ca(OEt)_2_-based nanocomposites depict enhanced bioactivity.

Confirmation of the formation of the HCA layer on the surfaces of the nanocomposites after SBF incubation was assessed using FTIR (Figure 6B) and XRD (Figure 6C). The FTIR spectra of nanocomposites presented peaks corresponding to the deformation vibration of PO_4_^3−^ ions at 567, 600 and 670 cm^−1^ [28]. The band at 600 cm^−1^ was mostly prominent in the CaCl_2_-containing nanocomposites, whereas the peak at 670 cm^−1^ was slightly more noticeable in the Ca(OEt)_2_-based nanocomposites. Some known bands overlapped with the peaks corresponding to the nanocomposites, but a distinguished peak was observed at around 960 relating to the deformation vibration of PO_4_^3−^ ions. In addition, further peaks corresponding to the CO_3_^2−^ functional group were observed. These peaks were observed at 1550 and 1460 cm^−1^ and were assigned to the A- and B-type carbonation (Figure 6B). A-type carbonation occurs when the carbonate ions replace the hydroxyl ions, while B-type carbonation appears when the carbonate ions enter the hydroxyapatite lattice replacing the phosphate ions [74]. In addition, a band at around 875 cm^−1^ was starting to form corresponding to the labile surface CO_3_^2−^ functional group [75,76,77]. The latter indicated the substitution of CO_3_^2−^ ions into the apatite, confirming the formation of HCA on the surface of nanocomposite hydrogels [28,75,77]. An increase in the peak intensity at around 800 cm^−1^, corresponding to the Si−O−Si vibration, was observed for all nanocomposites. The increased band intensity suggests the existence of a silica-rich layer [30]. Similarly, the asymmetric stretching of the siloxane bond (Si−O−Si) at around 1025 cm^−1^ became broader in the CaCl_2_-based 50-50-0 nanocomposite, which could also indicate the formation of a silica-rich layer. Moreover, a peak at 2980 cm^−1^ was observed due to the presence of the H−C−O functional group [77].

Further evaluation of the existence of mixed polycrystalline HCA layer on the surfaces of nanocomposites was assessed by XRD. The 50-50-1 nanocomposites were not evaluated since, according to Figure 6A,B, the bioactive properties of nanocomposites were not affected by the addition of MWCNTs in the gelatin–BG nanocomposites. Additionally, the 50-50-1 samples present the same chemical groups as their respective CaCl_2_ or Ca(OEt)_2_-containing 50-50-0 nanocomposites. Figure 6C shows the XRD data of the 50-50-0 control and SBF-treated nanocomposites composed of CaCl_2_ or Ca(OEt)_2_. Compared to the controls, which depicted an amorphous structure [45], nanocomposites treated in SBF presented diffraction peaks corresponding to the formation of HCA. These peaks were observed at 2θ = 27°, 31°, 45° and 56° which were associated with the diffraction planes (002), (211), (222), and (004) with reference to ICDD file #9-432 [18,28]. The crystalline peaks of the 50-50-0 containing Ca(OEt)_2_ were notably higher than the CaCl_2_-based 50-50-0. This could imply that the formation of crystallinity obtained from Ca and P depositions after SBF incubation was higher in the Ca(OEt)_2_-containing nanocomposites due to calcium ions in the silicate network. During incubation in SBF, the nanocomposites degrade through the exchange between Ca^2+^ ions from the glass component and the H^+^ ions from the SBF solution, causing hydrolysis of the silica groups to form silanol (Si−OH) and Si(OH)_4_ [8,37]. Condensation of the Si−OH groups subsequently occur, leaving a silica-rich layer on the surfaces of the nanocomposites [8]. The Ca^2+^ and PO_4_^3−^ groups then migrate to the silica-rich layer surface where they form a film of CaO–P_2_O_5_ which further crystallizes as the OH^−^ and CO_3_^2−^ anions from the SBF solution incorporate to form a mixed polycrystalline HCA layer on the surfaces of the nanocomposites [8]. Since the calcium ions in the 50-50-0 nanocomposite containing Ca(OEt)_2_ are incorporated in the glass network, the initial ion exchange between the Ca^2+^ ions from the BG and the H^+^ ions from the SBF is promoted leading to enhanced mineralization on the surfaces of Ca(OEt)_2_-based nanocomposites. In the case of CaCl_2_-containing nanocomposites, the calcium ions are leached out of the biomaterial and the rate of hydrolysis of the silica groups could potentially be reduced resulting in a decreased formation of mixed polycrystalline HCA layer and hence, lower intense crystalline peaks. Therefore, Figure 6 confirms that nanocomposites containing either CaCl_2_ or Ca(OEt)_2_ as calcium sources present bioactive properties and were higher for those containing Ca(OEt)_2_ due to the incorporation of calcium ions to the silicate glass network promoting hydrolysis by initial ion exchange. In addition, the bioactivity of nanocomposites was not affected by the incorporation of MWCNTs since mineralization was contributed by the presence of BG. Collectively, the bioactivity present in the 50-50-0 and 50-50-1 nanocomposites is a favorable property in bone biomaterials which could contribute to the potential development of new bone tissue. As stated in the introduction, this study aimed to evaluate the role of calcium sources (calcium chloride vs. calcium ethoxide) in a gelatin–BG system in terms of their physicochemical properties. We showed that the calcium ethoxide-based nanocomposites formed Si−O−NBO bridges as evidence for calcium incorporation into the silicate glass (Figure 1B). Furthermore, we demonstrated that the reduced mass loss is attributed to the network stability and lower porosity attributed to minimal leaching of the ions and gelatin (Figure 4B, Table 2). Nanocomposites based on Ca(OEt)_2_ had enhanced bioactivity in SBF indicating HCA formation compared with CaCl_2_ (Figure 6C, Table 3). Taken together, our results collectively demonstrated the superiority of the Ca(OEt)_2_ -based nanocomposites compared to CaCl_2_. However, calcium ion release studies which could have further strengthened the above-mentioned results, were not conducted. Future studies should include quantifying the released calcium ions. As a limitation of this study, further evaluations would have to be conducted to assess the thickness of the HCA layer on the surfaces of the nanocomposites to compare and confirm the robustness of the bioactivity of the different calcium-containing nanocomposites.

### 3.7. Mouse Embryo Multipotent Mesenchymal Progenitor 10T1/2 Cell Adhesion, Spreading and Viability on CaCl_2_- and Ca(OEt)_2_-Based Nanocomposites

**Figure 7:**
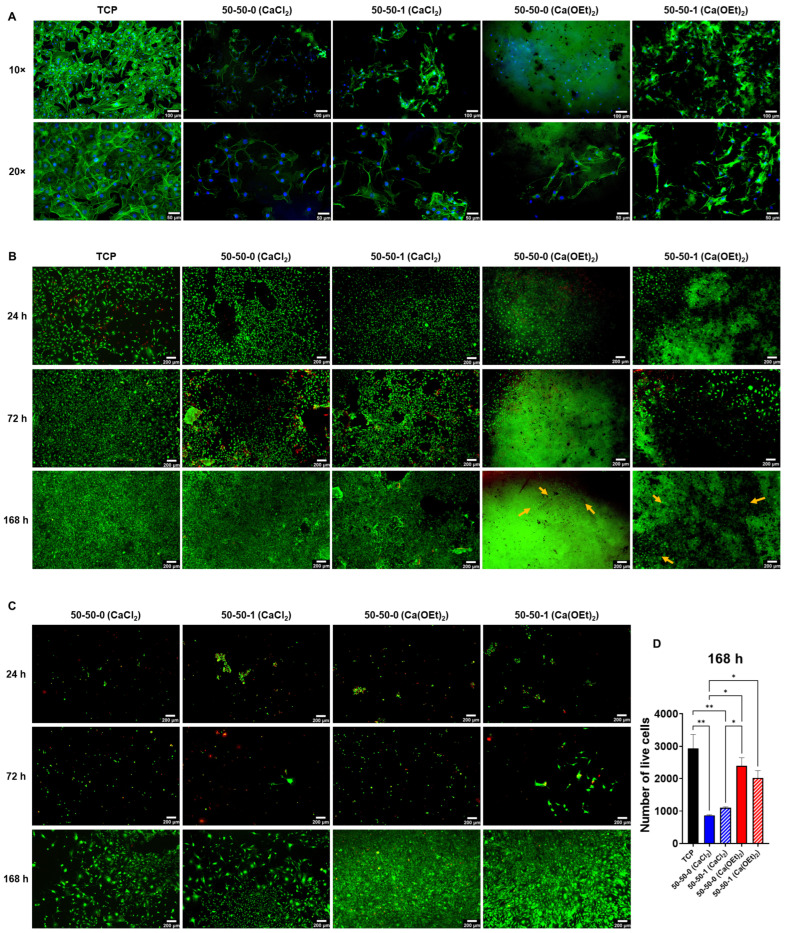
Attachment and viability of 10T1/2 cells on CaCl_2_- and Ca(OEt)_2_-based 50-50-0 and 50-50-1 nanocomposites. (**A**) Fluorescent images of 10T1/2 cells after 24 h of culture. Scale bar = 100 µm at 10× magnification and scale bar = 50 µm at 20× magnification. (**B**) Live/dead staining of 10T1/2 cells cultured on CaCl_2_- and Ca(OEt)_2_-based 50-50-0 and 50-50-1 nanocomposites. Scalebar = 200 µm. (**C**) Live/dead staining on cells cultured with conditioned media (extracts). Scalebar = 200 µm. (**D**) Number of live cells at 168 h of conditioned media culture. * *p* < 0.05, ** *p* < 0.01.

Cell-materials interaction studies were performed on the 50-50-0 and 50-50-1 nanocomposites prepared with CaCl_2_ and Ca(OEt)_2_. Figure 7A shows the fluorescent images of 10T1/2 cells cultured on a tissue culture plate (TCP) as control and on the surfaces of nanocomposites for 24 h. Cells had a favorable attachment and spreading to both CaCl_2_ and Ca(OEt)_2_-based nanocomposites by forming a uniform layer of elongated actin filaments onto the surfaces of the biomaterials. Entrapment of gelatin in the inorganic network could potentially act as an initial integrin recognition site for cells. This innate property of gelatin would be possible since it possesses an arginine-glycine-aspartic acid (RGD) sequence that could further favor the cell-ECM interactions, thus promoting cell adhesion [78]. Cells cultured on nanocomposites composed of CaCl_2_ showed a more elongated morphology than those composed of Ca(OEt)_2_. This could be due to the presence of micropores in the nanocomposites containing CaCl_2_, as previously discussed in the micro-CT results, which could improve initial cell attachment. Figure 7B–D show the viability of 10T1/2 cells cultured on the surfaces of nanocomposites using standard media (Figure 7B) or cultured on TCP using conditioned media (Figure 7C,D) for 24, 72, and 168 h accordingly. Cells cultured on TCP with standard media were used as control. The live/dead staining presented in Figure 7B shows that cells had good viability when cultured on the nanocomposites for different time points. Imaging cells cultured on Ca(OEt)_2_-based nanocomposites was challenging most notably at 168 h of culture as the biomaterials seemed to have been stained. However, viable 10T1/2 cells were observed and are indicated in orange arrows in the Ca(OEt)_2_-based 50-50-0 and 50-50-1 nanocomposites. In addition, it was observed that as the culture time increased, so did the viability of cells, which was comparable to that of TCP, especially by 168 h of culture. An analysis of the number of live cells was performed using conditioned media containing the extracts of nanocomposites (Figure 7C,D). 10T1/2 cells were not as viable at 24 h of culture with conditioned media, likely due to the calcium ions from the biomaterials. This was especially observed in the CaCl_2_-based nanocomposites since the calcium ions are physically bound to the matrix network, which can be easily diffused upon contact with the cell culture media during the conditioning process. The calcium ions released in the conditioned media could change the ionic strength, which can result in cell death [79]. The initial retained cells adapted to the conditioned media and started to grow between 72 and 168 h of culture. At 168 h of culture, 10T1/2 cells had adapted to the conditioned media containing extracts, and cell viability was favorable. Thus, cell density on cells cultured with conditioned media was higher at day 7. Although nanocomposites containing Ca(OEt)_2_ showed a decreased number of live cells compared to TCP, the difference in viable cells at 168 h of culture was not significant (Figure 7D). However, the number of viable cells on the CaCl_2_-based nanocomposites was considerably lower (*p* < 0.01) than TCP control. In addition, the 50-50-0 Ca(OEt)_2_ nanocomposite was significantly higher (*p* < 0.05) than the CaCl_2_-containing biomaterials, whereas the 50-50-1 Ca(OEt)_2_ was only substantially higher (*p* < 0.05) than the 50-50-0 CaCl_2_ nanocomposite.

The addition of MWCNTs to the gelatin–BG nanocomposites did not hinder the adhesion and viability of cells, which could enable future studies aimed at their effects on osteogenic cells. The incorporation of MWCNTs to organic–inorganic bone nanocomposites could not only mimic the endogenous electrically conductive properties of bone but could also potentially deliver electrical signals for the maturation of osseous tissue [42,43,44]. These results show that CaCl_2_- and Ca(OEt)_2_-based 50-50-0 and 50-50-1 nanocomposites could be used as potential bone biomaterials.

## 4. Conclusions

In this study, we prepared sol-gel-derived gelatin–BG–MWCNT nanocomposites using CaCl_2_ and Ca(OEt)_2_ in an attempt to evaluate the different calcium sources for the development of bone biomaterials with an electrically conductive component. The nanocomposites varied chemically and were dependent on the calcium source used for their preparation. Calcium ions from the Ca(OEt)_2_-based biomaterials were incorporated in the silicate glass nanocomposite network, whereas the calcium ions from CaCl_2_-containing biomaterials were not. However, the surface elemental distribution was homogeneous for both CaCl_2_- and Ca(OEt)_2_-containing nanocomposites. Furthermore, swelling and degradation of nanocomposites occurred as a function of time and were significantly different due to the fate of the calcium ions within the organic–inorganic network. The calcium ions from the calcium sources used also influenced the resulting porosity of nanocomposites post-degradation. The bioactivity of CaCl_2_- and Ca(OEt)_2_-based nanocomposites was demonstrated through the formation of hydroxycarbonate apatite on their surfaces after incubation in SBF for 7 days. Finally, 10T1/2 cells showed favorable adhesion and spreading on nanocomposites composed of CaCl_2_ and Ca(OEt)_2_ at 24 h of incubation. Cell viability studies using standard and conditioned media showed that Ca(OEt)_2_-based nanocomposites seemed more favorable biomaterials. Uniformly distributed MWCNTs did not hinder any of the properties of nanocomposites, which was a desired attribution to carry out future studies on their electrically conductive properties. Although Ca(OEt)_2_-based nanocomposites are most likely to have more advantageous properties in bone-repair applications, both CaCl_2_- and Ca(OEt)_2_-based nanocomposites have the potential to function as bone biomaterials to repair and regenerate osseous defects.

## Figures and Tables

**Table 1:** Nomenclature of gelatin–BG–MWCNT nanocomposites.

Gelatin-BG-MWCNT Nomenclature	Gelatin (wt.%)	BG (wt.%)	MWCNT (wt.%)
50-50-0	50	50	0
50-50-1	50	50	1

MWCNT was used as an additional filler and the weight percentage added was with respect to the total gelatin–BG composition, which is 100 wt.%.

**Table 2:** Pore properties and porosity of CaCl_2_- and Ca(OEt)_2_-containing nanocomposites.

Sample	Porosity (%)	Pore Wall Thickness (µm)	Pore Size (µm)	Surface Area to Volume Ratio (mm^−1^)
50-50-0 (CaCl_2_)	24.48	0.49	0.16	4.08
50-50-1 (CaCl_2_)	29.59	0.41	0.16	4.90
50-50-0 Ca(OEt)_2_	5.03	1.64	0.06	1.22
50-50-1 Ca(OEt)_2_	5.84	1.92	0.10	1.04

**Table 3:** Atomic percentages of Ca and P before and after SBF incubation including Ca/P ratio of CaCl_2_- and Ca(OEt)_2_-based nanocomposites after SBF treatment.

Sample	Atomic % Ca before SBF	Atomic % P before SBF	Atomic % Ca after SBF	Atomic % P after SBF	Ca/P Ratio after SBF
50-50-0 CaCl_2_	6.40	0.03	16.93	8.76	1.93
50-50-1 CaCl_2_	5.49	0.03	17.93	9.62	1.86
50-50-0 Ca(OEt)_2_	5.83	0.04	20.40	10.34	1.97
50-50-1 Ca(OEt)_2_	4.85	0.04	20.20	10.53	1.91

## Data Availability

The data that support the findings of this study are available from the corresponding author, Kibret Mequanint, upon reasonable request.
